# Making a low-cost retinal surgical simulator

**Published:** 2019-09-10

**Authors:** James Rice

**Affiliations:** 1 Vitreoretinal Consultant: University of Cape Town Division of Ophthalmology, Cape Town, South Africa.

**In surgical training, simulation offers an opportunity to practice skills away from the patient. This low-cost simulator has been developed for retinal surgery trainees.**

**Figure 1a:**
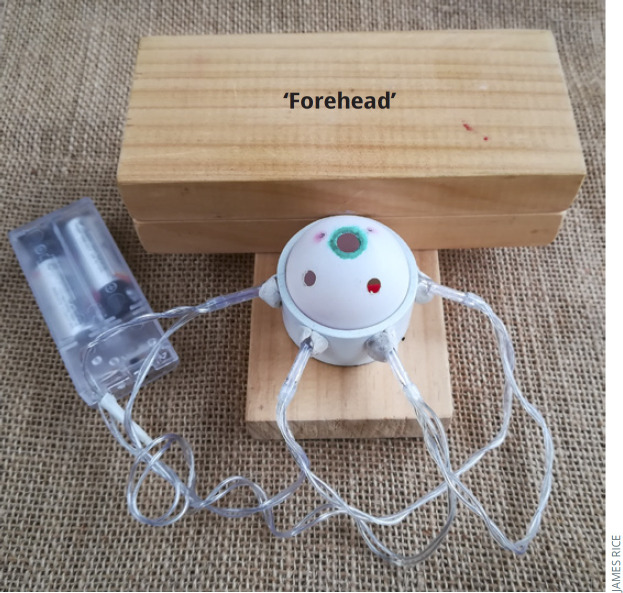
Completed model

New surgical skills should be learned in a safe, low-stress environment that does not put the patient at risk. Trying to learn new techniques while operating on patients increases the risk of complications and makes for an anxious learning experience for the trainee surgeon. It is also stressful for the trainer and, most importantly, the patient.

Surgical simulation provides a learning environment that poses no risk to the patient and puts the surgeon at ease. Several simulators are now available for training retinal surgeons. Unfortunately, access to this type of technology is limited in low- and middle-income countries because of cost. Similarly, artificial eyes for training are expensive and may have limited re-use. With this in mind, we have constructed a simple, very cheap eye model which is used under the retinal microscope to train surgical dexterity with retinal instruments. The cost of the model is less than USD $20 and it can be constructed in a few hours. The complete model is shown in Figure 1a.

## How to construct the base

What you will need (Figure 1b):

- Three pieces of wood for the base and ‘forehead’, each 15 cm × 7 cm × 2 cm.
- A plastic tube or pipe with 40 mm *internal* diameter, to act as the ‘eye socket’. 40 mm PVC pipe is too narrow, but a 40 mm PVC pipe connector works perfectly. This supports the ball and allows free rotation in all directions.
- Battery powered string lights (also known as ‘fairy lights’ or ‘Christmas tree lights’).
- Re-usable adhesive putty.

## Instructions

1. Using Figure 1b as a guide, glue two pieces of wood together to create the ‘forehead’ and use the other to create the platform for the support ring (‘eye socket’).
2. Cut the pipe connector to an appropriate length (about 22 mm). If you have a connector that has a central internal ‘ridge’, then I suggest cutting it 11 mm above the ridge and 8 mm below it (Figure 1c).
3. The top of the ball in the support ring should rest just below the level of the ‘forehead’ so that the hands are in the same position as during surgery.
4. Drill 5 mm holes 2–3 mm from the upper edge of the support ring where the lights will go (Figure 1c). We recommend at least three or four lights.
5. Glue the support ring in place.
6. The first three or four lights nearest the batteries are used. The rest of the lights can be cut off and the cut ends sealed with electrical tape. Place the lights in the drilled holes and hold in place using re-usable adhesive putty. They should not protrude through the holes so they don't affect rotation of the ball (Figure 1d). Alternatively, place the lights in holes in the ball (visible in Figure 1a); however, the weight of the wire tends to drag the ball.

**Figure 1b:**
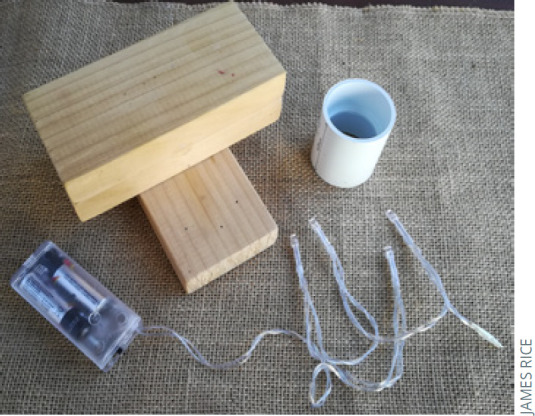


**Figure 1c:**
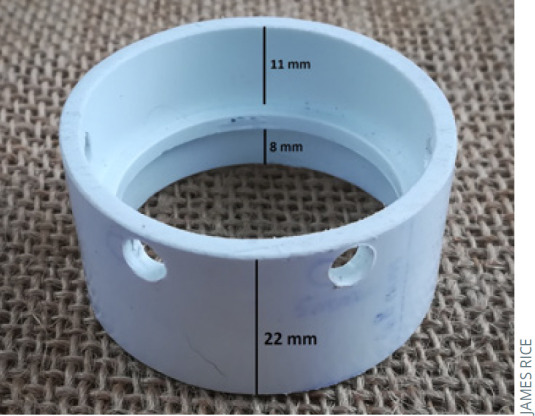


**Figure 1d:**
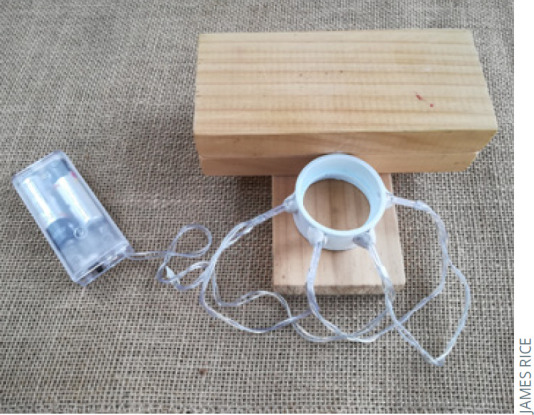


## How to construct the tasks in the balls (eyes)

What you will need (Figure 2a):

- Three standard white table tennis or ping-pong balls, 40 mm in diameter, that serve as the ‘eyes’. A different task is set up in each ball.
- One wooden and one sponge base (10 mm thick), filed or cut to fit inside the bottom section of the ball. These raise the floor of the ball, so that standard surgical instruments can reach the training tasks.
- A thin piece of non-stick silicone rubber cut from a ‘cookie’ or ‘muffin’ mould (available in some baking shops), the same size as the surface of the block of wood.
- A piece of pencil eraser/rubber and two sewing needles.
- Cotton thread.
- A piece of multi-strand electric wire.
- Re-usable adhesive putty.
- Spray-on membrane dressing. We used Opsite Spray®, which costs US $25 for 100 ml and makes multiple membranes.

## Instructions

1. Use a small drill or soldering iron (which easily melts the plastic) to create a 7 mm wide ‘pupil’ in each ping-pong ball. Draw the limbus in the approximate position around the pupil.
2. At 3.5 mm from the limbus, the normal anatomical position of the pars-plana, use an 18-gauge hypodermic needle to make small openings for the instruments. Enlarge these as necessary so that you feel no friction on the instruments.
3. Cut a wide slit (35 mm by 10 mm) in the side of the ball just below the ‘equator’ to insert the tasks (Figure 2b).
4. For ball 1 (Figure 3a), use a needle to string the cotton thread across the inside of the ball. Insert a sponge base.
5. For ball 2, use re-usable putty adhesive to fix the pencil eraser/rubber to the bottom of the ball. Insert the two sewing needles at a slight angle (Figure 3b).
6. For ball 3, place a thin, even layer of the putty adhesive on top of the wooden base. Insert the base and secure in place with more putty.
7. To prepare the membrane, spray a thin layer of the spray-on dressing onto the non-stick silicone rubber. Colour the membrane with a felt-tipped pen and use a razor blade to score it gently. The membrane peels effectively in strips which are easy to visualise. Unscored membrane is useful for bimanual tasks.
8. Slide the silicone rubber with prepared membrane gently through the side opening onto the top of the block and tap gently in place with a pencil through the ‘pupil’. The thin layer of putty will keep the silicone in place, but it can be easily removed to make a new membrane.

## Using the model

Place the model on the operating table and use the indirect viewing system attached to the operating microscope. The different balls are easily interchanged to perform the different tasks.

You can invent your own tasks that require fine motor movements. Useful tasks include:

- Balancing small loops of wire (cut from single strands of the multi-strand electric wire) onto the threads that have been strung across the inside of ball 1 (Figure 3a).
- Threading a wire through the eye of the needle using a single hand or as a two-handed exercise (Figure 3b).
- Cutting out small printed shapes (Figure 3c). You can do this in any of the balls.
- Manipulating a fine membrane (Figure 3d)
- Moving a loop along a wire maze with an indicator light (construction described elsewhere)^1^ (Figures 3e and 3f).

**Figure 2a:**
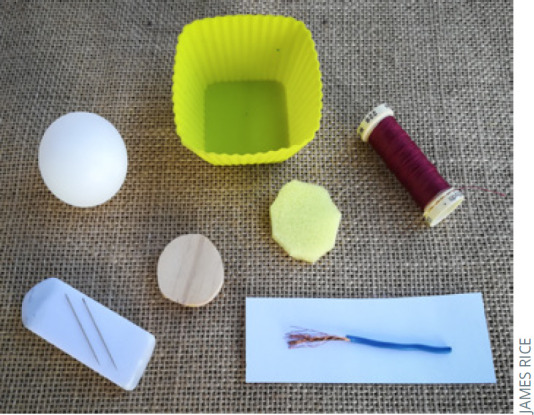


**Figure 2b:**
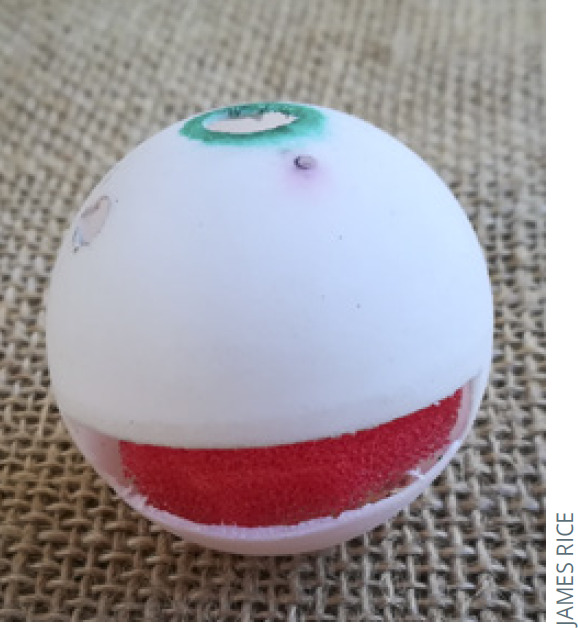


## Conclusion

Although the model does not teach specific procedures (such as retinal detachment repair), it does provide a realistic surgical environment. It allows the trainee to work with real retinal instruments and learn fine motor control. Our trainees have also found it useful for learning orientation with the indirect viewing system, including how to adjust field and magnification with the foot pedals, how to align the microscope with globe rotation and the effects of the instrument shaft on globe rotation.

The time to perform certain tasks improves with practice. If an assistant eyepiece is present, a supervisor can observe and teach instrument control.

A full description with videos is published elsewhere^2^. A formal validation study of the usefulness of the model as a training tool is being conducted.

**Figure 3a:**
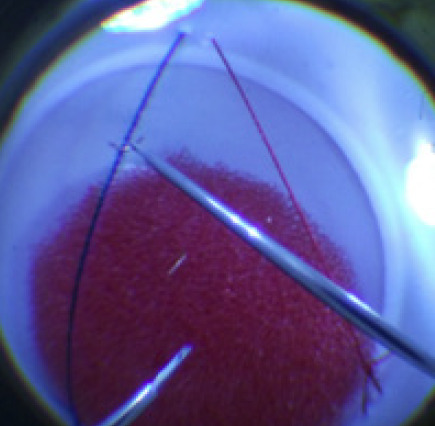
Wire loops on a cotton thread

**Figure 3b:**
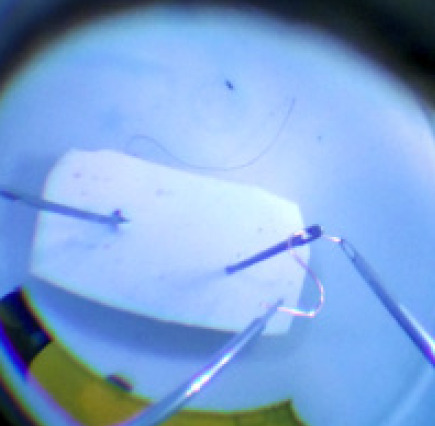
Wire loop through the eye of a needle

**Figure 3c:**
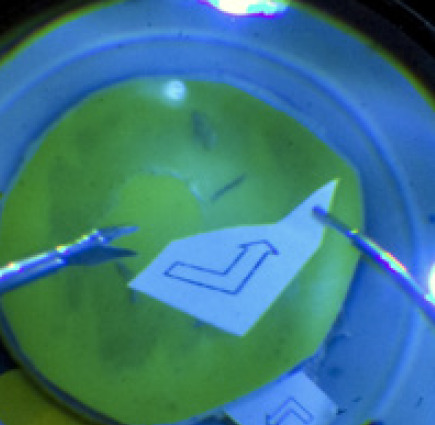
Cutting out printed shapes

**Figure 3d:**
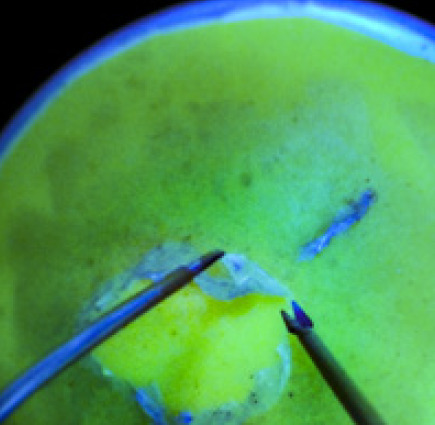
Manipulation of a fine membrane

**Figure 3e:**
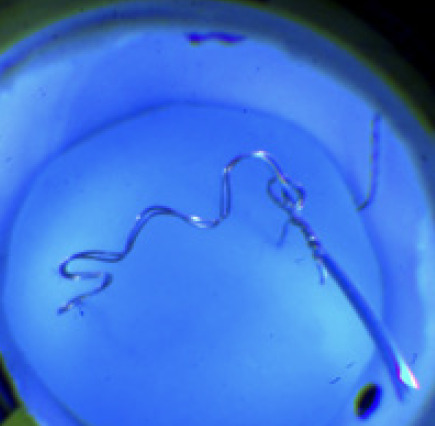
Wire loop without touch

**Figure 3f:**
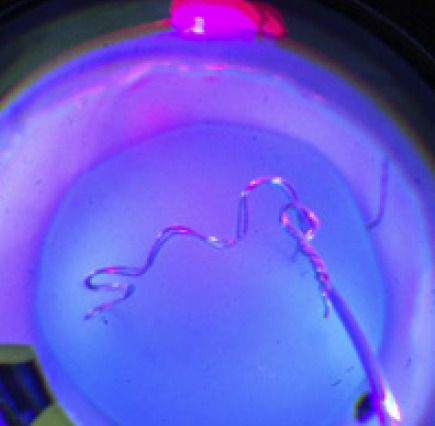
Wire loop with light indicating touch
